# First records of the fanged frogs *Limnonectes bannaensis* Ye, Fei & Jiang, 2007 and *L. utara* Matsui, Belabut & Ahmad, 2014 (Amphibia: Anura: Dicroglossidae) in Thailand

**DOI:** 10.3897/BDJ.9.e67253

**Published:** 2021-07-13

**Authors:** Chatmongkon Suwannapoom, Ke Jiang, Yun-He Wu, Parinya Pawangkhanant, Sengvilay Lorphengsy, Tan Van Nguyen, Nikolay A. Poyarkov, Jing Che

**Affiliations:** 1 Division of Fishery, School of Agriculture and Natural Resources, University of Phayao, Phayao, Thailand Division of Fishery, School of Agriculture and Natural Resources, University of Phayao Phayao Thailand; 2 CAS Key Laboratory of Mountain Ecological Restoration and Bioresource Utilization & Ecological Restoration and Biodiversity Conservation Key Laboratory of Sichuan Province, Chengdu Institute of Biology, Chinese Academy of Sciences, Chengdu, Sichuan, China CAS Key Laboratory of Mountain Ecological Restoration and Bioresource Utilization & Ecological Restoration and Biodiversity Conservation Key Laboratory of Sichuan Province, Chengdu Institute of Biology, Chinese Academy of Sciences, Chengdu Sichuan China; 3 State Key Laboratory of Genetic Resources and Evolution, Kunming Institute of Zoology, Chinese Academy of Sciences, Kunming, Yunnan, China State Key Laboratory of Genetic Resources and Evolution, Kunming Institute of Zoology, Chinese Academy of Sciences, Kunming Yunnan China; 4 Division of Biotechnology, School of Agriculture and Natural Resources, University of Phayao, Phayao, Thailand Division of Biotechnology, School of Agriculture and Natural Resources, University of Phayao Phayao Thailand; 5 Department of Species Conservation, Save Vietnam’s Wildlife,, Ninh Binh, Vietnam Department of Species Conservation, Save Vietnam’s Wildlife, Ninh Binh Vietnam; 6 Faculty of Biology, Department of Vertebrate Zoology, Moscow State University, Moscow, Moscow, Russia Faculty of Biology, Department of Vertebrate Zoology, Moscow State University, Moscow Moscow Russia; 7 Laboratory of Tropical Ecology, Joint Russian-Vietnamese Tropical Research and Technological Center, Hanoi, Vietnam Laboratory of Tropical Ecology, Joint Russian-Vietnamese Tropical Research and Technological Center Hanoi Vietnam

**Keywords:** Nan Province, Yala Province, 16S rRNA, Cryptic species, Species complex

## Abstract

**Background:**

The taxonomic status of the Thai populations belonging to the *Limnonectes kuhlii* species complex is controversial, due to phenotypic similarity in the cryptic species complex. Recently, some studies on this group in Thailand have discovered four new species: *L. taylori*, *L. megastomias*, *L. jarujini* and *L. isanensis*. Even so, the diversity of this group is still incomplete.

**New information:**

Based on an integrative approach encompassing genetic and morphological analyses, we conclude that the *Limnonectes* populations from Nan Province (northern) and Yala Province (southern) of Thailand are conspecific with *L. bannaensis* Ye, Fei & Jiang, 2007 and *L. utara* Matsui, Belabut & Ahmad, 2014, respectively. These are the first records of these species in Thailand. Our study highlights the importance of using DNA sequence data in combination with morphological data to accurately document species identity and diversity. This is especially important for morphologically cryptic species complexes and sympatrically occurring congeners.

## Introduction

*Limnonectes* Fitzinger, 1843 is the most species rich genus of Asian frogs of the family Dicroglossidae presently comprising 78 species distributed throughout East and Southeast Asia (Frost 2021). This genus is generally characterised by a morphological crypsis and contains several potentially undescribed cryptic species, especially within widespread species complexes, such as the *L. kuhlii* (Tschudi 1838) complex (McLeod 2010, Suwannapoom et al. 2016). In terms of a systematic framework, the *L. kuhlii* species complex in Thailand had been previously explored and four species are currently recorded within the country ([Bibr B6866771][Bibr B6866750], McLeod et al. 2012). According to established records, *L. megastomias* McLeod, 2008 is only found in Nakhon Ratchasima and Sa Kaeo Provinces of eastern Thailand. Two other species (*L. taylori* Matsui, Panha, Khonsue & Kuraishi, 2010 and *L. jarujini* Matsui, Panha, Khonsue & Kuraishi, 2010), occur in the mountain ranges of western Thailand, with *L. taylori* documented from northern and *L. jarujini* from southern parts of the country (Matsui et al. 2010a). Furthermore, *Limnonectes isanensis* McLeod, Kelly & Barley, 2012 is only known to occur in Phu Luang National Park, Loei Province, Thailand (McLeod et al. 2012).

During the course of our recent herpetological surveys conducted in the northern and southern parts of Thailand from 2017-2018, we collected several *Limnonectes* specimens from previously not examined populations in Nan and Yala Provinces. Based on detailed morphological comparisons and a phylogenetic analyses, we confirm here the presence of two *Limnonectes* species previously unreported for Thailand: *L. bannaensis* Ye, Fei, Xie & Jiang, 2007 and *L. utara* Matsui, Belabut & Ahmad, 2014, respectively. Therefore, we report *L. bannaensis* and *L. utara* as two new records of amphibian species for Thailand, provide morphological and morphometric descriptions of the collected specimens and remark on the natural history of these species, based on our field observations.

## Materials and methods

### Sampling

**Figure 1.**
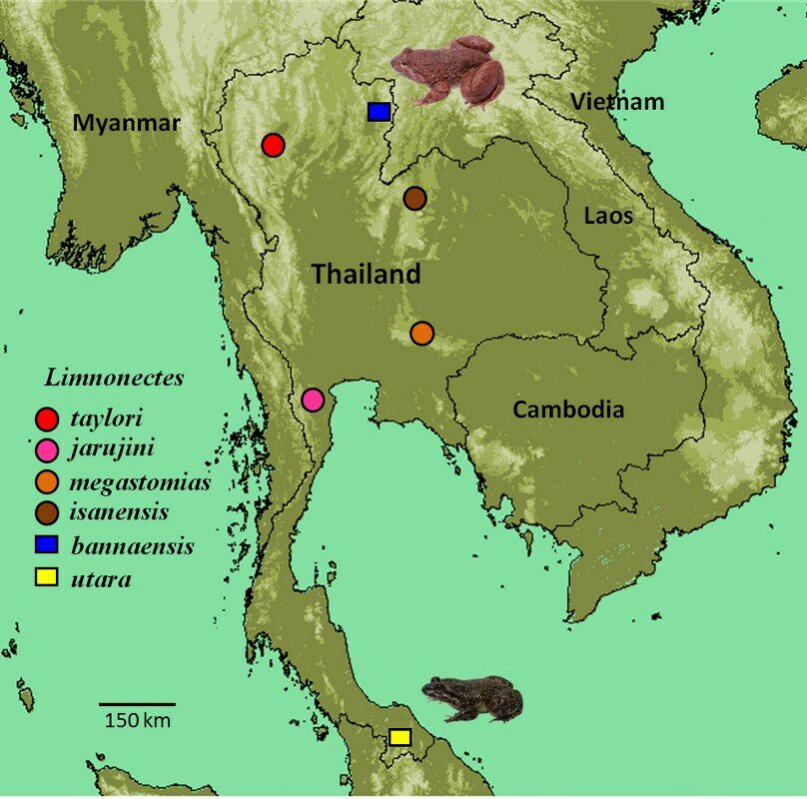
Map showing the type-localities of *Limnonectes kuhlii* species complex in Thailand. Circles = Type localities of *L. taylori* (red), *L. jarujini* (purple), *L. megestomias* (orange) and *L. isanensis* (brown). Blue square (*L. bannaensis*) and yellow square (*L. utara*) represent the two new distribution records in Thailand reported here.

Field surveys were conducted in Bo Kluea District, Nan Province in December 2017, in Bannang Sata District, Yala Province in August 2018 (Fig. 1). A total of 11 specimens of *Limnonectes bannaensis* and four specimens of *L. utara* were collected. Liver tissue samples of all specimens were taken and preserved in 95% ethanol for molecular analysis. The specimens were fixed with 10% formalin for 24 hours and subsequently transferred to 70% ethanol. Specimens and tissues were subsequently deposited at the herpetological collections of the School of Agriculture and Natural Resources, University of Phayao (AUP), Phayao, Thailand.

### Molecular analysis

Genomic DNA was extracted from liver tissues preserved in 95% ethanol using the standard phenol-chloroform extraction protocol (Sambrook et al. 1989). Partial fragments of the mitochondrial 16S rRNA were amplified for all samples via the polymerase chain reaction (PCR) using the following primers: 16SAR (5'-CGCCTGTTTAYCAAAAACAT-3') and 16SBR (5'-CCGGTYTGAACTCAGATCAYGT-3'). PCR amplifications were performed in a 25 µl reaction volume with the following cycling conditions: initial denaturing step at 95°C for 4 min, 35 cycles of denaturing at 94°C for 40 s, annealing at 55°C for 30 s, extending at 72°C for 1 min and a final extension step at 72°C for 10 min. PCR products were directly sequenced using an ABI 3730xl DNA automated sequencer with both forward and reverse primers.

Matrilineal genealogies were reconstructed to examine genealogical relationships amongst *Limnonectes*, based on the 16S rRNA gene fragment. Homologous sequences of the related species of *Limnonectes* and those of the outgroups (*Fejervarya limnocharis* (Gravenhorst) and *Fejervarya iskandari* Veith, Kosuch, Ohler & Dubois were downloaded from GenBank (see Table 1).

Trees were reconstructed using Bayesian Inference (BI) and Maximum Likelihood (ML). JMODELTEST v.2.1.7 (Darriba et al. 2012) was used to select an appropriate nucleotide substitution model for BI. The GTR+G model was chosen as the best-fit model following the Bayesian Information Criterion (BIC, Posada 2008). The CIPRES web server (Miller et al. 2010) was selected to implement BI. The Monte Carlo Markov chain length was run for 10,000,000 generations and sampled every 1,000 generations. A burn-in value of 25% was used. Convergence was assessed by the average standard deviation of split frequencies (below 0.01) and the ESS values (over 200) in TRACER v.1.5 (Rambaut A and Drummond A 2007). ML was performed using RAxML with 1,000 bootstrap replicates (Stamatakis et al. 2008).

### Morphometric analysis and morphological comparisons

Morphometric measurements were taken using digital callipers to the nearest 0.1 mm, following Matsui (1984) and McLeod 2008) abbreviations of the morphometric traits are as follows: snout-vent length (SVL), horizontal eye diameter (ED), eye nostril distance (END), rostrum length distance (RLD), thigh (femur) length (FEL), foot length (FOL), head length (HL), head width (HW), internarial distance (IN), interorbital width (IO), lower arm length (LAL), mandible-nostril distance (MN), palm length (PAL), relative finger length (RFL), relative toe length (RTL), shank (tibia) length (TBL), tympanum diameter (TD) and upper eyelid width (UEW). The digital-webbing formulae followed Savage (1975). Morphological comparisons were made with specimens of morphologically related congeners, deposited at the University of Phayao (AUP).

## Data resources

### Molecular phylogeny

**Figure 2.**
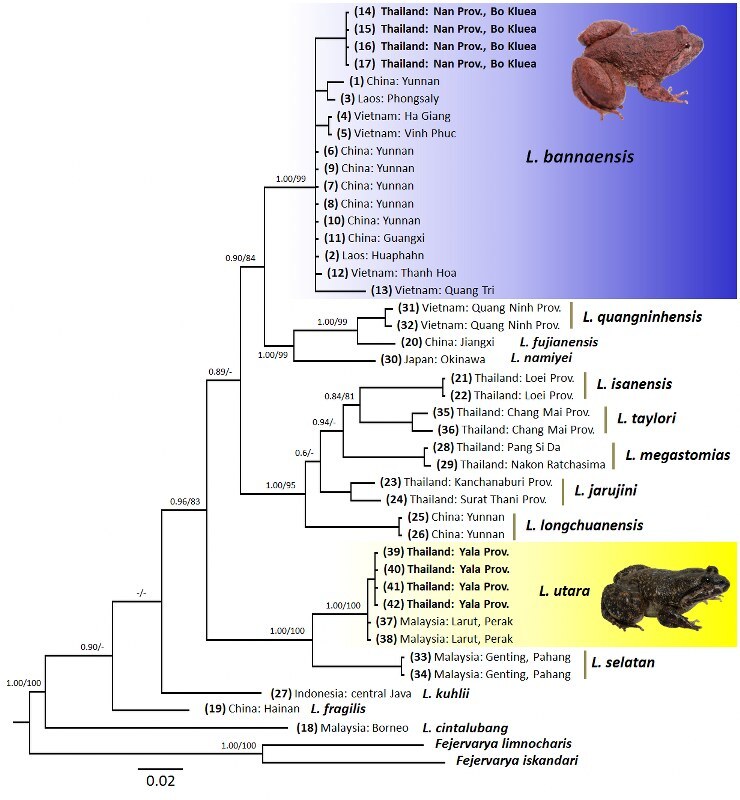
BI tree resulting from 492 bp length fragment of mitochondrial 16S rRNA gene for *Limnonectes* species and outgroups. Bayesian posterior probabilities (BPP) > 95%/ML inferences (ML-BS) > 80% are shown for each node; “-” denotes low support of Bayesian posterior probabilities and bootstrap support < 80% in one analysis, no values on branches represent low support in both analyses. The scale bar represents 0.02 nucleotide substitutions per site.

Sequencing generated a total of 492 base pairs (bp) of 16S rRNA for *Limonectes bannaensis* and *L. utara*. All newly-generated sequences were submitted to GenBank (Accession numbers MZ493344-MZ493351, see Table 1). Interspecific uncorrected p-distances between the newly-discovered population of *L. bannaensis* collected from Nan Province in Thailand and the other known species of *Limnonectes* varied from 6.3% (in relation to *L. quangninhensis*) to 12.1% (in relation to *L. cintalubang*) (Suppl. material 1). The uncorrected p-distance between the newly-found populations of *L. bannaensis* from Nan Province and the topotypic *L. bannaensis* (Mengyang, Yunnan, China) is 2.1%. Both ML and BI analyses recovered the Nan population nested within a strongly supported clade, together with topotypic *L. bannaensis* (see Fig. 2). The newly-discovered population of *L. utara* from Yala Province, Thailand and the congeners varied from 5.8% (in relation to *L. selatan*) to 13.5% (in relation to *L. cintalubang*) (see Suppl. material 1). The uncorrected p-distance between the newly-discovered populations of *L. utara* from Yala Province and the topotypic *L. utara* (Larut, Perak, Malaysia) is 0.2%. Both ML and BI analyses recovered the Yala population within a strongly supported clade, together with topotypic L. utara (Fig. 2).

## Taxon treatments

### Limnonectes bannaensis

Ye, Fei, Xie & Jiang, 2007

37820612-A313-589D-A03D-6F890EE0127B

#### Materials

- **Type status:** Other material. **Occurrence:** catalogNumber: AUP-00481; individualCount: 1; sex: male; lifeStage: adult; **Taxon:** scientificName: *Limnonectes bannaensis*; class: Amphibia; order: Anura; family: Dicroglossidae; genus: Limnonectes; specificEpithet: *bannaensis*; scientificNameAuthorship: Ye, Fei, Xie & Jiang, 2007; **Location:** country: Thailand; countryCode: TL; stateProvince: Nan; locality: Doi Phu Kha; verbatimElevation: 750; verbatimLatitude: 19°03'21.3"N; verbatimLongitude: 101°10'47.8"E; **Event:** eventDate: 17 December, 2017; fieldNotes: collected by C. Suwannapoom, P. Pawangkhanant; **Record Level:** language: en; collectionCode: Amphibians; basisOfRecord: Preserved Specimen
- **Type status:** Other material. **Occurrence:** catalogNumber: AUP-00482; individualCount: 1; sex: adult male; **Taxon:** scientificName: *Limnonectes bannaensis*; **Record Level:** basisOfRecord: Preserved Specimen; dynamicProperties: collection date, collector and Location as the AUP-00481
- **Type status:** Other material. **Occurrence:** catalogNumber: AUP-00483; individualCount: 1; sex: adult male; **Taxon:** scientificName: *Limnonectes bannaensis*; **Record Level:** basisOfRecord: Preserved Specimen; dynamicProperties: collection date, collector and Location as the AUP-00481
- **Type status:** Other material. **Occurrence:** catalogNumber: AUP-00484; individualCount: 1; sex: adult male; **Taxon:** scientificName: *Limnonectes bannaensis*; **Record Level:** basisOfRecord: Preserved Specimen; dynamicProperties: collection date, collector and Location as the AUP-00481
- **Type status:** Other material. **Occurrence:** catalogNumber: AUP-00485; individualCount: 1; sex: adult male; **Taxon:** scientificName: *Limnonectes bannaensis*; **Record Level:** basisOfRecord: Preserved Specimen; dynamicProperties: collection date, collector and Location as the AUP-00481
- **Type status:** Other material. **Occurrence:** catalogNumber: AUP-00486; individualCount: 1; sex: adult male; **Taxon:** scientificName: *Limnonectes bannaensis*; **Record Level:** basisOfRecord: Preserved Specimen; dynamicProperties: collection date, collector and Location as the AUP-00481
- **Type status:** Other material. **Occurrence:** catalogNumber: AUP-00487; individualCount: 1; sex: adult male; **Taxon:** scientificName: *Limnonectes bannaensis*; **Record Level:** basisOfRecord: Preserved Specimen; dynamicProperties: collection date, collector and Location as the AUP-00481
- **Type status:** Other material. **Occurrence:** catalogNumber: AUP-00488; individualCount: 1; sex: adult male; **Taxon:** scientificName: *Limnonectes bannaensis*; **Record Level:** basisOfRecord: Preserved Specimen; dynamicProperties: collection date, collector and Location as the AUP-00481
- **Type status:** Other material. **Occurrence:** catalogNumber: AUP-00489; individualCount: 1; sex: adult female; **Taxon:** scientificName: *Limnonectes bannaensis*; **Record Level:** basisOfRecord: Preserved Specimen; dynamicProperties: collection date, collector and Location as the AUP-00481
- **Type status:** Other material. **Occurrence:** catalogNumber: AUP-00490; individualCount: 1; sex: adult male; **Taxon:** scientificName: *Limnonectes bannaensis*; **Record Level:** basisOfRecord: Preserved Specimen; dynamicProperties: collection date, collector and Location as the AUP-00481
- **Type status:** Other material. **Occurrence:** catalogNumber: AUP-00491; individualCount: 1; sex: adult female; **Taxon:** scientificName: *Limnonectes bannaensis*; **Record Level:** basisOfRecord: Preserved Specimen; dynamicProperties: collection date, collector and Location as the AUP-00481

#### Description

**Figure 3.**
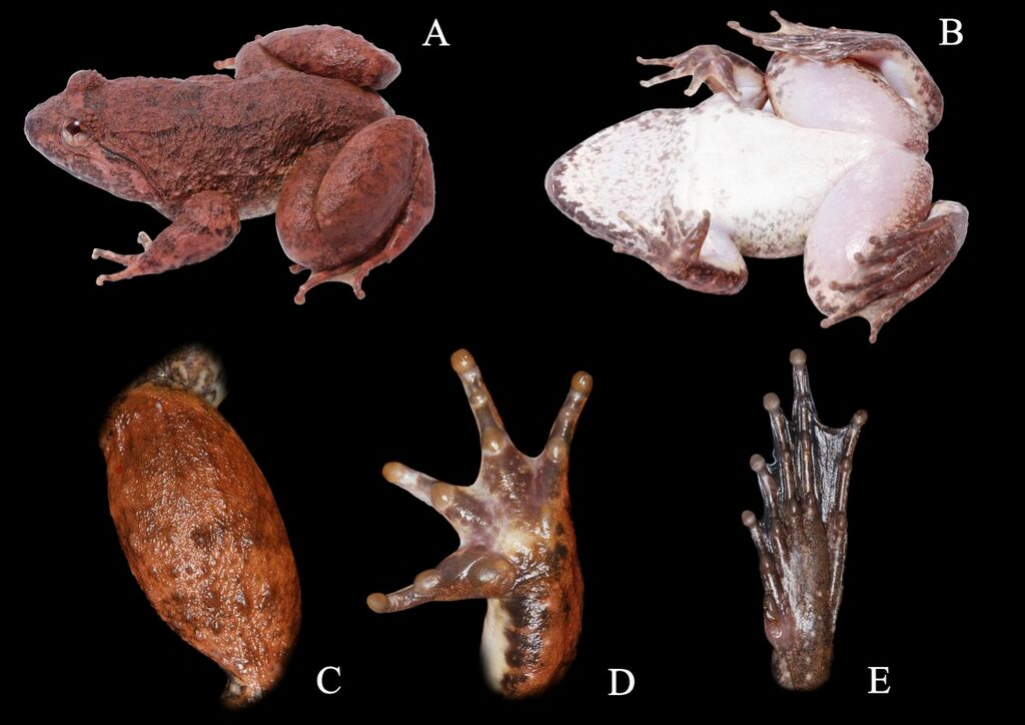
Male of *Limnonectes bannaensis* (AUP-00485) in life. **A.** Dorsal view; **B.** Ventral views; **C.** Dorsal view of leg (notice the tubercles); **D.** Palmar view of hand; **E.** Ventral view of foot.

**Figure 4.**
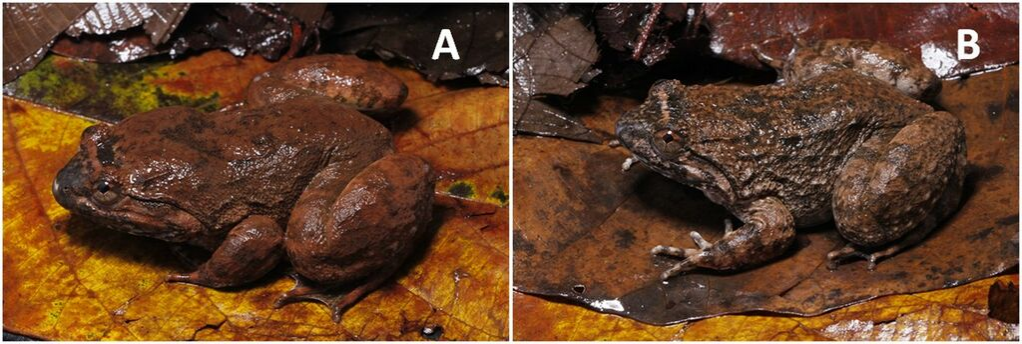
Colour variations of *Limnonectes bannaensis* **A.** Dorsal view of male (AUP-00481); **B.** Dorsal view of female (AUP-00491).

Morphological characters of specimens from Nan Province agreed with the descriptions by Ye et al. (2007). Large body size, with males SVL of 80.7 mm (n = 9) and females SVL of 75.4 mm (n = 2). The complete morphometric description of each specimen is presented in Suppl. material 2. They are morphologically distinct in comparison between sexes. Males can be distinguished from females by the dorsal skin texture of the male appearing to be smoother, with less tubercles, supratympanic fold dark brown, indistinct, throat heavily pigmented. Head longer than wide (males HL of 36.8 mm, HW 34.9 mm, n = 9 and females HL of 34.9 mm, HW 32.9 mm, n = 2). Fore limbs robust, relatively short, fingers moderately slender, finger length formula: II< I < IV < III (Fig. 3D), toe length formula: I < II < V < III < IV (Fig. 3E), tips of toes expanded into round elevated pads lacking grooves, toe webbing well-developed, complete, webbing formula: I 0 – 0 II 0 – 0 III 0 – 0 IV 0 – 0 V. Skin on dorsum weakly granulated with few fine folds on the back and a few small rounded tubercles scattered on the rear of the dorsum, ventrally smooth. Colouration in life: black stripes present on areas around the folds (Fig. 3A and C), dorsum light red brown with confluent dark brown markings (Fig. 3A and B and Fig. 4), dark transverse bars on upper surface of hind limbs, side of head and lateral surfaces of body lighter brown, lower lip white marbled with brown, belly white with brown vermiform markings, dark brown bar between eyes edged with thin yellowish-brown bars, lower half of iris golden, upper half brown, separated by a dark brown horizontal band, nuptial pad white. Colouration in preservative: after three years in preservative, the colouration pattern did not change, dorsal and lateral body colouration faded to brown, dark brown bars on upper lip turned less distinct, lower lip turned dark with light mottling, ventre immaculate, ventral portions of limbs mottled around margins, palmar and plantar surfaces turned dark brown.

#### Distribution

This species is known from southern China, northern and central Vietnam and northern Laos (Frost 2020). This is the first record for Thailand, ca. 266 km southwest from the type locality in Jinghong City, Mengla County, Yunnan Province, China (Ye et al. 2007).

#### Ecology

**Figure 5.**
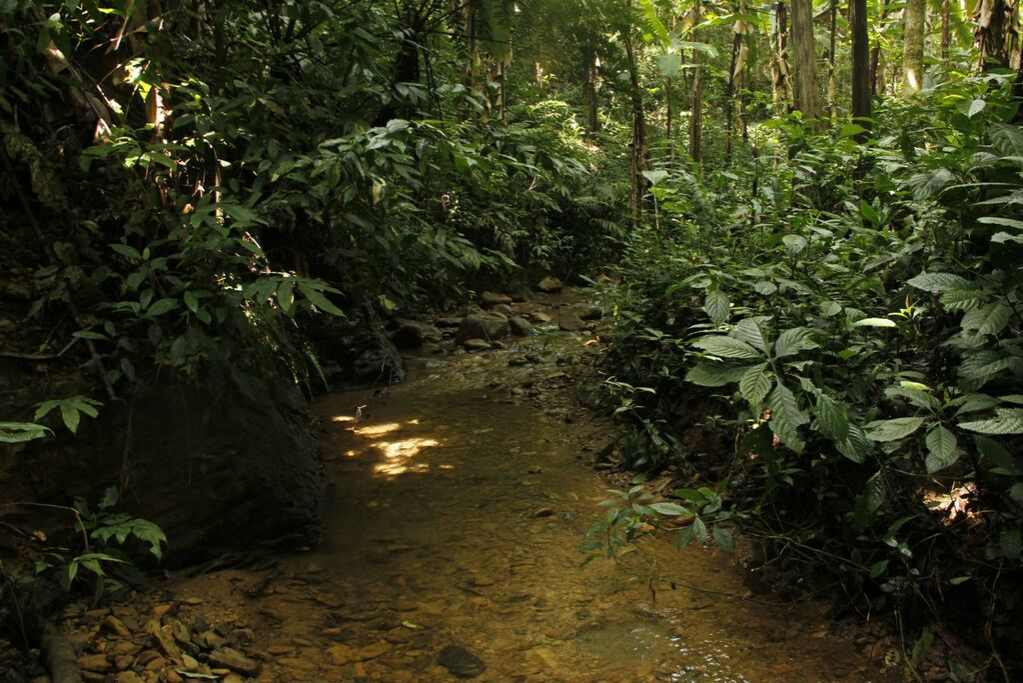
Habitat of *Limnonectes bannaensis* in Bo Kluea District, Nan Province, northern Thailand.

Specimens were found between 19:00 to 21:00 h in small rocky streams (Fig. 5). Most specimens were found in the water. The surrounding habitat was secondary evergreen forest of medium growth. Other anuran species found in sympatry include: *Limnonectes taylori*, *Kurixalus bisacculus* (Taylor), Leptobrachium cf. huashen Fei & Ye, Leptobrachella cf. minima (Taylor) and *Amolops cremnobatus* Inger & Kottelat.

### Limnonectes utara

Matsui, Belabut & Ahmad, 2014

3AB4F5C4-4351-5BFE-BA79-AEBF06DC6DB9

#### Materials

- **Type status:** Other material. **Occurrence:** catalogNumber: AUP-01706; individualCount: 1; sex: female; lifeStage: adult; **Taxon:** scientificName: *Limnonectes utara*; class: Amphibia; order: Anura; family: Dicroglossidae; genus: Limnonectes; specificEpithet: *utara*; scientificNameAuthorship: Matsui, Belabut & Ahmad, 2014; **Location:** country: Thailand; countryCode: TL; stateProvince: Yala; locality: Bannang Sata; verbatimElevation: 680; verbatimLatitude: 6°11'39.5"N; verbatimLongitude: 101°18'28.2"E; **Event:** eventDate: 21 August, 2018; fieldNotes: P. Pawangkhanant, C. Suwannapoom; **Record Level:** language: en; collectionCode: Amphibians; basisOfRecord: Preserved Specimen
- **Type status:** Other material. **Occurrence:** catalogNumber: AUP-01706; individualCount: 1; sex: female; lifeStage: adult; **Taxon:** scientificName: *Limnonectes utara*; **Record Level:** basisOfRecord: Preserved Specimen; dynamicProperties: collection date, collector and Location as the AUP-01705
- **Type status:** Other material. **Occurrence:** catalogNumber: AUP-01707; individualCount: 1; sex: male; lifeStage: adult; **Taxon:** scientificName: *Limnonectes utara*; **Record Level:** basisOfRecord: Preserved Specimen; dynamicProperties: collection date, collector and Location as the AUP-01705
- **Type status:** Other material. **Occurrence:** catalogNumber: AUP-01708; individualCount: 1; sex: male; lifeStage: adult; **Taxon:** scientificName: *Limnonectes utara*; **Record Level:** basisOfRecord: Preserved Specimen; dynamicProperties: collection date, collector and Location as the AUP-01705

#### Description

**Figure 6.**
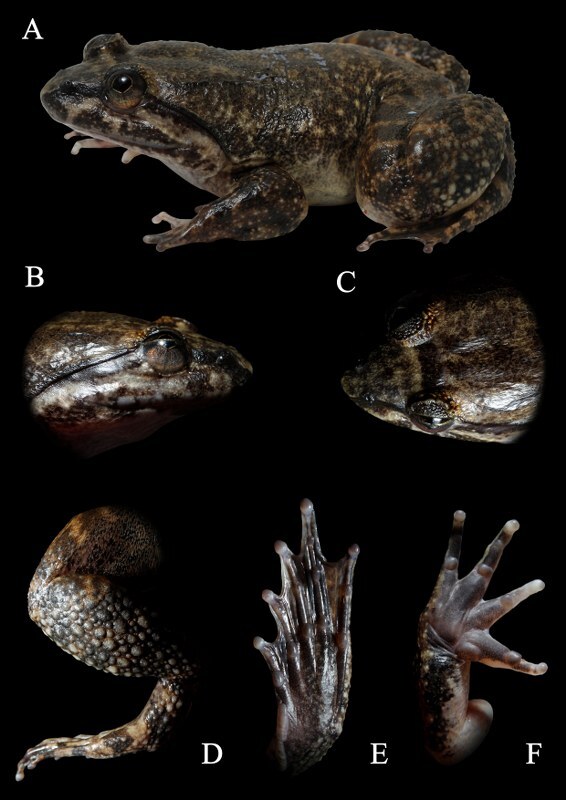
Male of *Limnonectes utara* (AUP-01708) in life. **A.** Dorsal view; **B.** Lateral view of head; **C.** Dorsolateral view of head; **D.** Dorsal view of leg (notice the tubercles); **E.** Ventral view of foot; **F.** Ventral view of hand.

Morphological characters of specimens from Yala Province agreed with the description by Matsui et al. (2014): Body size moderate, with males SVL of 70.7 mm (n = 2) and females SVL of 46.1 mm (n = 2). The complete morphometric description of each specimen is presented in Suppl. material 2. Head slightly longer than wide (males HL of 32.8 mm, HW 29.8 mm, n = 2 and females HL of 20.0 mm, HW 19.6 mm, n = 2). Snout obtusely pointed in dorsal view, obtuse in profile, projecting beyond the lower jaw. Eye diameter shorter than snout length, canthus rostralis rounded, loreal region sloping and concave, nostril dorsolaterally orientated, placed closer to tip of snout than to eye, internarial distance equal to upper eyelid width. Fore limb robust, relatively short and moderately slender fingers, finger length formula: II< I < IV < III (Fig. 6F), toe length formula, I < II < V < III < IV (Fig. 6E), tips of toes expanded into round, elevated pads lacking grooves, toe webbing complete, webbing formula, I 0 – 0 II 0 – 0 III 0 – 0 IV 0 – 0 V.

Skin on dorsal surfaces of head, fore limbs and body feebly crenulate, skin of body flanks rough with moderately, roundish and non-pearl tipped tubercles, skin around vent, knees and shanks distinctly tuberculate, covered with moderately, small, low tubercles with translucent spinules, ventral surfaces smooth, pair of faint, but broken dorsolateral folds extending from posterior of eye to vent.

Colouration in life: dorsum light brown with confluent dark brown markings (Fig. 6A), head with narrow light bands placed anteriorly to the dark interorbital bar, blackish-brown stripe on canthus rostralis, sides of head pale brown with dark markings. Ventral surfaces of hand and foot dark brown (Fig. 6E and F). Colouration in preservative: after two years in preservative, dorsal colouration slightly faded, but other than that, no obvious change in colour pattern has occurred.

#### Distribution

Prior to these records, this species was considered endemic to Peninsular Malaysia. This is the first country record for Thailand, ca. 158 km northeast from the type locality [Bukit Larut (= Larut Hill), Perak State, Peninsular Malaysia] (Matsui et al. 2014).

#### Ecology

**Figure 7.**
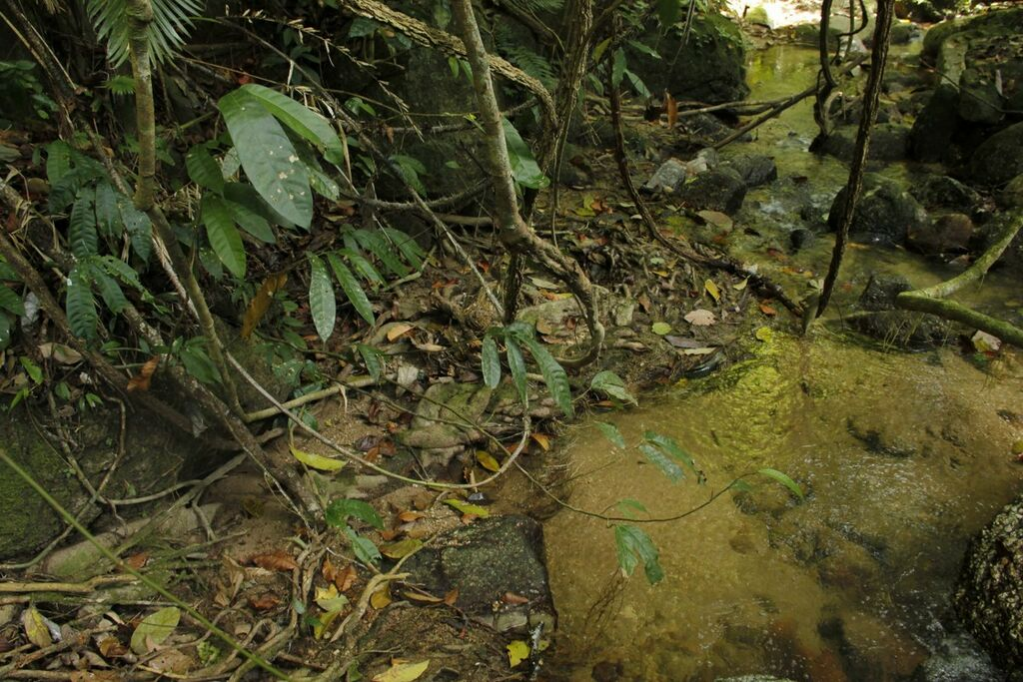
Habitat of *Limnonectes utara* in Bannang Sata District, Yala Province, southern Thailand.

Specimens were found after 20:00 h in small rocky streams. Most specimens were found in the water. All specimens were collected in evergreen forests along hillside streams and small tributaries varying in width from 1 m to 2 m (Fig. 7). Other syntopic anuran species include: *Limnonectes plicatellus* (Stoliczka), *Nyctixalus pictus* (Peters) and *Rhacophorus rhodopus* Liu & Hu.

## Discussion

In this study, we examined newly-collected samples of *Limnonectes* species related to the *L. kuhlii* species complex, from previously not surveyed areas in northern and southern Thailand. From a biogeographic perspective, according to Matsui et al. (2010a), *L. taylori* was thought to be the unique representative of the *L. kuhlii* species complex in northern Thailand, whereas *L. jarujini* was believed to occur in the southern part of the country, the biogeographic distribution between these species being located between Thong Pha Phum and Khao Laem National Parks in Kanchanaburi Province. With the exception of *L. bannaensis* and *L. utara*, it was already known which other species of the *L. kuhlii* complex occur in northern and southern Thailand; therefore, it is not a result that can be obtained from phylogenetic analysis. Actually, phylogeny corroborates the identification of the collected specimens and, thus, demonstrates that *L. bannaensis* and *L. utara* occur in northern and southern Thailand, respectively. Our new records of *L. bannaensis* and *L. utara* from Thailand increase to 20 the number of *Limnonectes* species occurring in the country.

Our study and others like this (e.g. Suwannapoom et al. 2016) further highlight the importance of using molecular data in combination with traditional morphological characteristics. This is especially important for species complexes whose members have sympatric distribution, which is the case with the *L. kuhlii* complex. We recorded sympatric occurrence of *L. taylori* and *L. bannaensis*, which were observed sharing the same habitats at Bo Kluea, Nan Province, northern Thailand. Consistent with the findings of previous studies involving the *Limnonectes* species complex (e.g. Suwannapoom et al. 2016), our results demonstrate that species living in sympatry are not necessarily close relatives (i.e. sister taxa).

These two sympatric members of the *Limnonectes* species complex in Bo Kluea, Nan Province, are difficult to distinguish from each other, based only on morphological evidence. The application of molecular methods is crucial for reliable identification and can guide morphological re-examinations, further elucidating fine-scale differences in morphological characteristics that represent species-specific variations. Identification of tadpoles, juveniles and adult females still remains challenging in the field. Our study underscores that the herpetofaunal diversity of Thailand still remains underestimated and also illustrates the special role of evergreen forests with regard to biodiversity conservation in the country.

## Supplementary Material

CD17D084-91A3-5CAC-A1E7-EA100936AEAF 10.3897/BDJ.9.e67253.suppl1 Supplementary material 1 Genetic distance between species of *Limnonectes*. Data type Table Brief description The pairwise uncorrected p-distance (%) of 16S rRNA gene between species of *Limnonectes*. File: oo_530422.docx https://binary.pensoft.net/file/530422 Chatmongkon Suwannapoom

D43584EA-6FD9-5678-BAC0-33BDB6E49F68 10.3897/BDJ.9.e67253.suppl2 Supplementary material 2 Measurement (in mm) and proportions of the series of *Limnonectes bannaensis* and *L. utara* Data type Table Brief description Measurements (in mm) and proportions of the series of *Limnonectes bannaensis* from Nan Province and *L. utara* from Yala Province. (M = Male, F = Female; N/a = Not applicable; for other abbreviations, see Materials and Methods). File: oo_562504.docx https://binary.pensoft.net/file/562504 Chatmongkon Suwannapoom

XML Treatment for Limnonectes bannaensis

XML Treatment for Limnonectes utara

## Figures and Tables

**Table 1.** Sequences and voucher specimens of *Limnonectes* and outgroup taxa used in molecular analyses for this study with sampling localities.

#	**Species**	**Voucher**	**Locality**	**GenBank**	**Reference**
1	*L. bannaensis*	CIB 200901116	China, Yunnan, Jinghong	AB526312	Matsui et al. 2010
2	*L. bannaensis*	FMNH 255140	Laos, Huaphahn, Vieng Tong	HM067133	McLeod 2010
3	*L. bannaensis*	FMNH 258519	Laos, Phongsaly, Phongsaly	HM067158	McLeod 2010
4	*L. bannaensis*	VNMN A.2015.41	Vietnam, Ha Giang, Vi Xuyen	HM067246	McLeod 2010
5	*L. bannaensis*	AMNH 106430	Vietnam, Vinh Phuc, Tam Dao	HM067272	McLeod 2010
6	*L. bannaensis*	KIZ 024971	China, Yunnan, Xishuangbanna, Mengla, Yiwu	KU599847	Suwannapoom et al. 2016
7	*L. bannaensis*	KIZ 024970	China, Yunnan, Xishuangbanna, Mengla, Yiwu	KU599848	Suwannapoom et al. 2016
8	*L. bannaensis*	KIZ 011793	China, Yunnan, Xishuangbanna, Mengla, Bubang	KU599849	Suwannapoom et al. 2016
9	*L. bannaensis*	KIZ 011726	China, Yunnan, Xishuangbanna, Mengyang	KU599850	Suwannapoom et al. 2016
10	*L. bannaensis*	KIZ 011727	China, Yunnan, Xishuangbanna, Mengyang	KU599851	Suwannapoom et al. 2016
11	*L. bannaensis*	KIZ 022207	China, Guangxi, Hulong, Pinglongshan	KU599856	Suwannapoom et al. 2016
12	*L. bannaensis*	KIZ 011608	Vietnam, Thanh Hoa, Quan Hoa	KU599857	Suwannapoom et al. 2016
13	*L. bannaensis*	KIZ YPX18365	Vietnam, Quang Tri, Bac Huong Hoa	KU599861	Suwannapoom et al. 2016
14	*L. bannaensis*	AUP-00481	Thailand, Nan, Bo Kluea	MZ493348	This study
15	*L. bannaensis*	AUP-00484	Thailand, Nan, Bo Kluea	MZ493349	This study
16	*L. bannaensis*	AUP-00485	Thailand, Nan, Bo Kluea	MZ493350	This study
17	*L. bannaensis*	AUP-00488	Thailand, Nan, Bo Kluea	MZ493351	This study
18	*L. cintalubang*	KUHE 47859	Malaysia, Borneo, Sarawak, Serian	AB981409	Matsui et al. 2010a
19	*L. fragilis*	CIB 20081089	China, Hainan, Wuzhi Shan	AB526315	Matsui et al. 2010
20	*L. fujianensis*	CIB ZJ 200806223	China, Jiangxi, Zixi	AB526311	Matsui et al. 2010
21	*L. isanensis*	KUHE 19284	Thailand, Loei, Phu Luang	AB526314	Matsui et al. 2010
22	*L. isanensis*	KUHE 19320	Thailand, Loei, Phu Luang	AB558955	Matsui et al. 2010a
23	*L. jarujini*	KUHE 19514	Thailand, Kanchanaburi, Sangkhla Buri	AB558940	Matsui et al. 2010a
24	*L. jarujini*	KUHE 19690	Thailand, Surat Thani, Khlong Saeng	AB558950	Matsui et al. 2010a
25	*L. longchuanensis*	KIZ048424	China, Yunnan, Dehong, Longchuan	KU599867	Suwannapoom et al. 2016
26	*L. longchuanensis*	KIZ048527	China, Yunnan, Yingjiang, Tongbiguan	KU599869	Suwannapoom et al. 2016
27	*L. kuhlii*	GMU unnumbered	Indonesia, Java, Purwerojo	AB526316	Matsui et al. 2010
28	*L. megastomias*	FMNH 266221	Thailand, Sa Kaew, Pang Si Da	HM067184	McLeod 2010
29	*L. megastomias*	KU 307760	Thailand, Nakon Ratchasima	HM067201	McLeod 2010
30	*L. namiyei*	KUHE L0809191	Japan, Okinawa, Okinawajima	AB526309	Matsui et al. 2010
31	*L. quangninhensis*	IEBR 3969	Vietnam, Quang Ninh, Hai Ha	KY595927	Pham et al. 2017
32	*L. quangninhensis*	IEBR 3970	Vietnam, Quang Ninh, Hai Ha	KY595928	Pham et al. 2017
33	*L. selatan*	KUHE54079	Malaysia, Genting, Pahang	AB981384	Matsui et al. 2010
34	*L. selatan*	KUHE54080	Malaysia, Genting, Pahang	AB981385	Matsui et al. 2010
35	*L. taylori*	KUHE 19101	Thailand, Chiang Mai, Doi Inthanon	AB558929	Matsui et al. 2010a
36	*L. taylori*	KUHE 19868	Thailand, Chiang Mai, Tha Ton	AB981390	Matsui et al. 2010a
37	*L. utara*	KUHE54064	Malaysia, Larut, Perak	AB981377	Matsui et al. 2010
38	*L. utara*	KUHE54065	Malaysia, Larut, Perak	AB981378	Matsui et al. 2010
39	*L. utara*	AUP 01705	Thailand, Yala, Bannang Sata	MZ493344	This study
40	*L. utara*	AUP 01706	Thailand, Yala, Bannang Sata	MZ493345	This study
41	*L. utara*	AUP 01707	Thailand, Yala, Bannang Sata	MZ493346	This study
42	*L. utara*	AUP 01708	Thailand, Yala, Bannang Sata	MZ493347	This study
**Outgroup**					
43	*F. limnocharis*	AMNH A-161230	Vietnam, Nghe An, Con Cuong, Pu Mat	AY843588	Faivovich et al. 2005
44	*F. iskandari*	UI unnumbered	Indonesia, Java, Banyuwangi	AB526324	Matsui et al. 2010
