# Supplementary material for: First records of the fanged frogs Limnonectes bannaensis Ye, Fei & Jiang, 2007 and L. utara Matsui, Belabut & Ahmad, 2014 (Amphibia: Anura: Dicroglossidae) in Thailand
Source: Biodivers Data J. 2021 Jul 13;9:e67253. doi: 10.3897/BDJ.9.e67253 (PMC8292287; doi:10.3897/BDJ.9.e67253)
Supplement: Supplementary material 1 — Genetic distance between species of Limnonectes. [file bdj-09-e67253-s001.docx]

**Table 2.**

The pairwise uncorrected p-distance (%) of 16S rRNA gene between species of *Limnonectes*.

|  | **Species** | 1 | 2 | 3 | 4 | 5 | 6 | 7 | 8 | 9 | 10 | 11 | 12 | 13 | 14 |
| --- | --- | --- | --- | --- | --- | --- | --- | --- | --- | --- | --- | --- | --- | --- | --- |
| 1 | ***L. utara* (AUP-017705)** |  |  |  |  |  |  |  |  |  |  |  |  |  |  |
| 2 | *L. selatan* | 5.8 |  |  |  |  |  |  |  |  |  |  |  |  |  |
| 3 | ***L. bannaensis* (AUP-00481)** | 9.0 | 9.6 |  |  |  |  |  |  |  |  |  |  |  |  |
| 4 | *L. namiyei* | 8.8 | 10.0 | 6.5 |  |  |  |  |  |  |  |  |  |  |  |
| 5 | *L. fujianensis* | 9.4 | 9.2 | 6.5 | 6.7 |  |  |  |  |  |  |  |  |  |  |
| 6 | *L. isanensis* | 10.6 | 11.3 | 8.3 | 9.6 | 9.4 |  |  |  |  |  |  |  |  |  |
| 7 | *L. jarujini* | 10.0 | 10.6 | 7.3 | 8.1 | 7.3 | 5.4 |  |  |  |  |  |  |  |  |
| 8 | *L. taylori* | 10.2 | 11.3 | 7.5 | 8.8 | 8.8 | 5.4 | 5.2 |  |  |  |  |  |  |  |
| 9 | *L. megastomias* | 10.6 | 11.9 | 7.9 | 8.5 | 9.2 | 5.6 | 5.4 | 5.2 |  |  |  |  |  |  |
| 10 | *L. fragilis* | 9.4 | 10.0 | 8.5 | 9.2 | 8.5 | 9.8 | 9.6 | 10.2 | 7.9 |  |  |  |  |  |
| 11 | *L. kuhlii* | 9.4 | 10.4 | 8.1 | 9.6 | 10.0 | 8.3 | 9.0 | 9.0 | 8.8 | 7.7 |  |  |  |  |
| 12 | *L. cintalubang* | 13.5 | 14.0 | 12.1 | 14.8 | 14.0 | 13.8 | 13.1 | 14.2 | 13.5 | 10.4 | 11.3 |  |  |  |
| 13 | *L. longchuanensis* | 10.6 | 9.6 | 8.3 | 7.9 | 8.1 | 7.3 | 5.6 | 6.9 | 6.5 | 9.6 | 9.6 | 14.0 |  |  |
| 14 | *L. quangninhensis* | 9.2 | 9.2 | 6.3 | 6.3 | 2.7 | 9.8 | 8.5 | 9.2 | 8.8 | 7.7 | 9.6 | 13.5 | 9.4 |  |
