# Supplementary material for: First records of the fanged frogs Limnonectes bannaensis Ye, Fei & Jiang, 2007 and L. utara Matsui, Belabut & Ahmad, 2014 (Amphibia: Anura: Dicroglossidae) in Thailand
Source: Biodivers Data J. 2021 Jul 13;9:e67253. doi: 10.3897/BDJ.9.e67253 (PMC8292287; doi:10.3897/BDJ.9.e67253)
Supplement: Supplementary material 2 — Measurement (in mm) and proportions of the series of Limnonectes bannaensis and L. utara [file bdj-09-e67253-s002.docx]

**Table 3.**

Measurement (in mm) and proportions of the series of *Limnonectes bannaensis* from Nan Province and *L. utara* from Yala Province. (M= Male, F= Female; N/a= Not applicable; for other abbreviations see Materials and methods).

| **Species** | ***Limnonectes bannaensis*** | | | | | | | | | | | | |
| --- | --- | --- | --- | --- | --- | --- | --- | --- | --- | --- | --- | --- | --- |
| Specimen | AUP 00481 | AUP 00482 | AUP 00483 | AUP 00484 | AUP 00485 | AUP 00486 | AUP 00487 | AUP 00488 | AUP 00490 | **Mean**  **(n = 9)** | AUP 00489 | AUP 00491 | **Mean**  **(n = 2)** |
| Sex | M | M | M | M | M | M | M | M | M | **M** | F | F | **F** |
| SVL | 92.4 | 86.7 | 85.6 | 87.9 | 83.2 | 77.5 | 71.5 | 72.4 | 69.2 | **80.7** | 74.6 | 76.2 | **75.4** |
| ED | 10 | 10.3 | 10.1 | 9.7 | 10.5 | 9.4 | 8.9 | 8.6 | 8.3 | **9.5** | 7.5 | 7.6 | **7.6** |
| END | 7.6 | 5.3 | 5.4 | 7.6 | 6.4 | 5.6 | 5.1 | 5.8 | 5.1 | **6.0** | 5.9 | 5.3 | **5.6** |
| RLD | 12.8 | 10.2 | 10.9 | 11.7 | 12 | 11.5 | 9.9 | 10.4 | 9.9 | **11.0** | 10.8 | 10 | **10.4** |
| FEL | 47.6 | 45.8 | 46 | 46.5 | 43.3 | 40.8 | 36.2 | 39.6 | 36 | **42.4** | 40.3 | 39.6 | **40.0** |
| FOL | 57.6 | 52.3 | 55.6 | 58.5 | 53.3 | 49.3 | 46.3 | 47.2 | 44.3 | **51.6** | 50.3 | 47.3 | **48.8** |
| HL | 41.7 | 37.8 | 40.3 | 40.6 | 38.4 | 36.7 | 32.4 | 34.4 | 29.1 | **36.8** | 36.2 | 33.5 | **34.9** |
| HW | 39.7 | 36.7 | 36.2 | 39.7 | 33.1 | 32.8 | 29.1 | 29.8 | 26 | **33.7** | 34.4 | 31.4 | **32.9** |
| IN | 7.9 | 6.8 | 8.2 | 7.1 | 6.4 | 6.9 | 5.7 | 6.5 | 5.2 | **6.7** | 5.3 | 6.0 | **5.7** |
| IO | 9.5 | 7.7 | 8.4 | 9.4 | 8.7 | 7.6 | 7.0 | 7.1 | 5.3 | **7.9** | 6.7 | 7.0 | **6.9** |
| LAL | 15.9 | 15.9 | 14.8 | 15.1 | 13.9 | 12.5 | 11.7 | 14 | 11.4 | **13.9** | 14.1 | 12.9 | **13.5** |
| MN | 35.6 | 36.6 | 35.7 | 36.2 | 38.4 | 33.1 | 28.9 | 33.5 | 29.6 | **34.2** | 32.8 | 27.0 | **29.9** |
| PAL | 23.5 | 21.1 | 22.7 | 23.6 | 21.0 | 20.8 | 18.7 | 18.8 | 17.8 | **20.9** | 18.9 | 17.5 | **18.2** |
| TBL | 43.9 | 43.1 | 42.2 | 42.6 | 38.4 | 49 | 34.4 | 46.2 | 33.7 | **41.5** | 37.4 | 35.8 | **36.6** |
| TD | N/a | N/a | N/a | N/a | N/a | N/a | N/a | N/a | N/a | **N/a** | N/a | N/a | **N/a** |
| UEW | 6.7 | 6.6 | 6.1 | 6.1 | 5.7 | 5.8 | 5.5 | 5.2 | 5.2 | **5.9** | 5.1 | 5.0 | **5.1** |
| ED/HL | 0.3 | 0.3 | 0.3 | 0.2 | 0.3 | 0.3 | 0.3 | 0.3 | 0.3 | **0.3** | 0.2 | 0.3 | **0.3** |
| HW/HL | 1.0 | 1.0 | 0.9 | 1.0 | 0.9 | 0.9 | 0.9 | 0.9 | 0.9 | **0.9** | 1.0 | 0.9 | **1.0** |
| HL/SVL | 0.5 | 0.4 | 0.5 | 0.5 | 0.5 | 0.5 | 0.5 | 0.5 | 0.4 | **0.5** | 0.5 | 0.4 | **0.5** |
| IN/IO | 0.8 | 0.9 | 1.0 | 0.8 | 0.7 | 0.9 | 0.8 | 0.9 | 1.0 | **0.9** | 0.8 | 0.9 | **0.9** |
| IN/SVL | 0.1 | 0.1 | 0.1 | 0.1 | 0.1 | 0.1 | 0.1 | 0.1 | 0.1 | **0.1** | 0.1 | 0.1 | **0.1** |
| END/IN | 1.1 | 0.8 | 0.7 | 1.1 | 1.0 | 0.8 | 0.9 | 0.9 | 1.0 | **0.9** | 1.1 | 0.9 | **1.0** |
| UEW/IO | 0.7 | 0.9 | 0.7 | 0.7 | 0.7 | 0.8 | 0.8 | 0.7 | 1.0 | **0.8** | 0.8 | 0.7 | **0.8** |
| END/RLD | 0.6 | 0.5 | 0.5 | 0.7 | 0.5 | 0.5 | 0.5 | 0.6 | 0.5 | **0.5** | 0.6 | 0.5 | **0.6** |
| TBL/SVL | 0.5 | 0.5 | 0.5 | 0.5 | 0.5 | 0.6 | 0.5 | 0.6 | 0.5 | **0.5** | 0.5 | 0.5 | **0.5** |
| FEL/SVL | 0.5 | 0.5 | 0.5 | 0.5 | 0.5 | 0.5 | 0.5 | 0.6 | 0.5 | **0.5** | 0.5 | 0.5 | **0.5** |
| LAL/SVL | 0.2 | 0.2 | 0.2 | 0.2 | 0.2 | 0.2 | 0.2 | 0.2 | 0.2 | **0.2** | 0.2 | 0.2 | **0.2** |

| **Species** | ***Limnonectes utara*** | | | | | |
| --- | --- | --- | --- | --- | --- | --- |
| Specimen | AUP  01707 | AUP  01708 | **Mean**  **(n = 2)** | AUP  01705 | AUP  01706 | **Mean**  **(n = 2)** |
| Sex | M | M | **M** | F | F | **F** |
| SVL | 64.1 | 77.3 | **70.7** | 46.4 | 45.7 | **46.1** |
| ED | 8.7 | 8.5 | **8.6** | 7.1 | 6.9 | **7.0** |
| END | 4.8 | 5.7 | **5.3** | 2.6 | 3.0 | **2.8** |
| RLD | 10.1 | 11.9 | **11.0** | 6.8 | 7.1 | **7.0** |
| FEL | 30.2 | 34.8 | **32.5** | 23 | 22 | **22.5** |
| FOL | 43.8 | 45.3 | **44.6** | 29.7 | 30.1 | **29.9** |
| HL | 30.8 | 34.7 | **32.8** | 19.6 | 20.4 | **20.0** |
| HW | 27.0 | 32.5 | **29.8** | 19.4 | 19.7 | **19.6** |
| IN | 4.8 | 5.2 | **5.0** | 3.9 | 3.4 | **3.7** |
| IO | 5.6 | 6.1 | **5.9** | 2.7 | 3.1 | **2.9** |
| LAL | 10.9 | 14.4 | **12.7** | 7.7 | 7.2 | **7.5** |
| MN | 25.2 | 30 | **27.6** | 17.2 | 17.3 | **17.3** |
| PAL | 15.9 | 18.9 | **17.4** | 10.8 | 11.9 | **11.4** |
| TBL | 29.5 | 34.7 | **32.1** | 21.6 | 21.9 | **21.8** |
| TD | 4.3 | 4.9 | **4.6** | 3.3 | 2.9 | **3.1** |
| UEW | 4.3 | 4.4 | **4.4** | 3.3 | 2.9 | **3.1** |
| ED/HL | 0.3 | 0.3 | **0.3** | 0.4 | 0.3 | **0.4** |
| HW/HL | 0.9 | 0.9 | **0.9** | 1.0 | 1.0 | **1.0** |
| HL/SVL | 0.5 | 0.5 | **0.5** | 0.4 | 0.5 | **0.5** |
| IN/IO | 0.9 | 0.9 | **0.9** | 1.4 | 1.1 | **1.3** |
| IN/SVL | 0.1 | 0.1 | **0.1** | 0.1 | 0.1 | **0.1** |
| END/IN | 1.0 | 1.1 | **1.1** | 0.7 | 0.9 | **0.8** |
| UEW/IO | 0.8 | 0.7 | **0.8** | 1.2 | 0.9 | **1.1** |
| END/RLD | 0.5 | 0.5 | **0.5** | 0.4 | 0.4 | **0.4** |
| TBL/SVL | 0.5 | 0.5 | **0.5** | 0.5 | 0.5 | **0.5** |
| FEL/SVL | 0.5 | 0.5 | **0.5** | 0.5 | 0.5 | **0.5** |
| LAL/SVL | 0.2 | 0.2 | **0.2** | 0.2 | 0.2 | **0.2** |
